# Jean Paul Euzéby (1949–2025)

**DOI:** 10.1099/ijsem.0.007014

**Published:** 2025-12-19

**Authors:** Aharon Oren, Markus Göker, Edward R.B. Moore

**Affiliations:** 1 The Institute of Life Sciences, The Hebrew University of Jerusalem, The Edmond J. Safra Campus, 9190401 Jerusalem, Israel; 2 Leibniz Institute DSMZ – German Collection of Microorganisms and Cell Cultures, Inhoffenstrasse 7B, 38124 Braunschweig, Germany; 3 Department of Infectious Disease and Culture Collection University of Gothenburg (CCUG), Institute for Biomedicine, Sahlgrenska Academy, University of Gothenburg, SE-402 34 Gothenburg, Sweden

**Keywords:** List Editor, Nomenclature, Obituary

**Figure:**
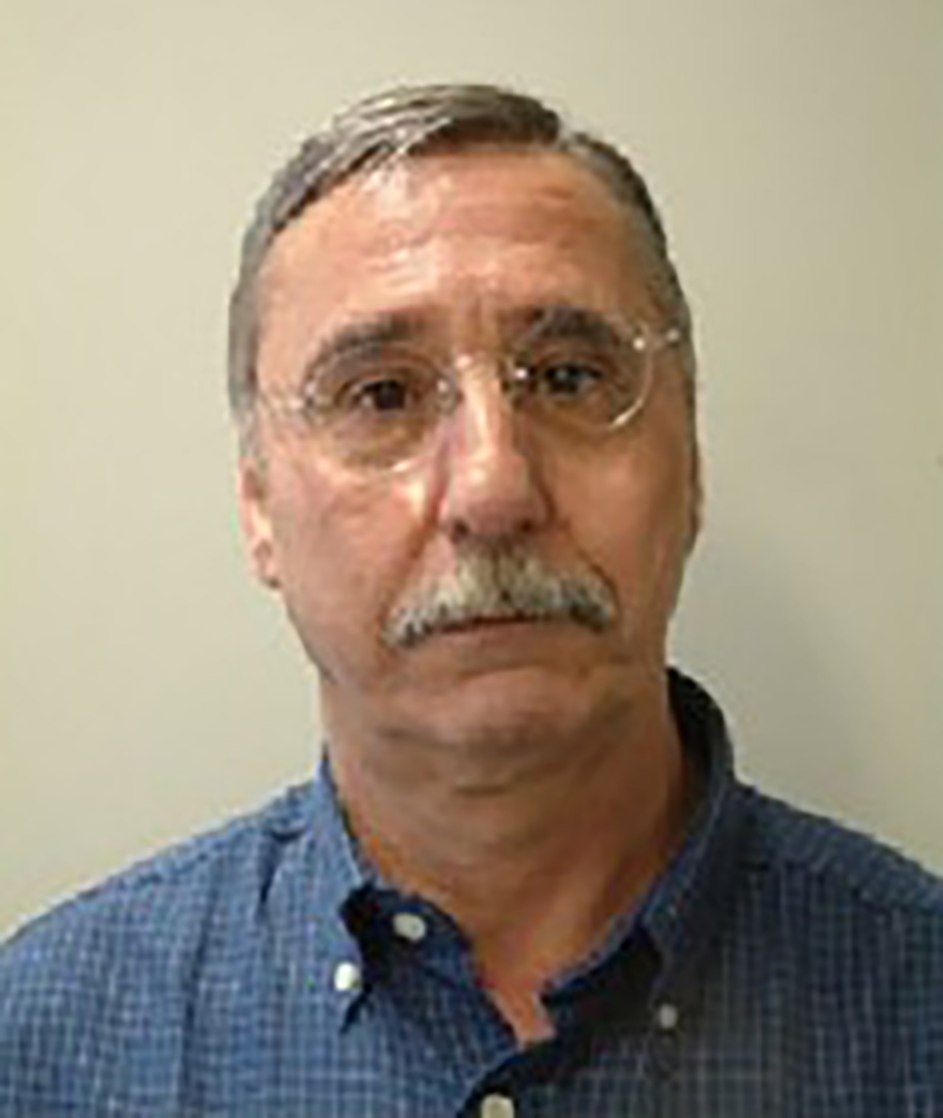


On 27 October 2025, Jean Paul Euzéby passed away in Toulouse, France. He was born on 22 February 1949, in Nîmes, France, the son of parasitologist Jacques Achille Marie Euzéby (1920–2010) and Renée Gayte (1921–2015).

Jean spent most of his career as a Professor of Microbiology at the École Nationale Vétérinaire de Toulouse (ENVT), where he was highly regarded by his students (and was even once elected Best Teacher at ENVT). He had also been employed for years in the ENVT library. To help students and everyone interested in bacteriology, he published an online dictionary of bacteriology. This website, updated on a daily basis until his retirement, was very much appreciated by the whole community.

In addition to his profession as a microbiologist, he was an accomplished linguist with a deep knowledge of classical Latin and Greek. This made him eminently qualified to establish order in nomenclature matters and orthography. He served as a Nomenclature Reviewer for the International Journal of Systematic and Evolutionary Microbiology (IJSEM) and, with the participation and assistance of his wife, Beatrice, checked all proposed names of new taxa, suggesting corrections where necessary and interacting with authors and editors to find the orthographically correct and most elegant solutions to all nomenclature problems. Jean was always happy to assist authors who consulted with him prior to the submission of their manuscripts. Together with the late Hans Georg Trüper, he revised and extended the orthography appendix (Appendix 9) of the *International Code of Nomenclature of Prokaryotes* [1]. In addition, he proposed many emendations of the *Code* and submitted Requests for Opinions to the International Committee on Systematics of Prokaryotes’ (ICSP) Judicial Commission.

Jean served as the List Editor of the IJSEM from 2003 until 2013, and during this period, he produced Validation Lists no. 91 (May 2003) to 152 (July 2013), Notification Lists from vol. 53, part 1 (May 2003) to vol. 65, part 5 (August 2013) and Lists of Changes in Taxonomic Opinion no. 1 (January 2005) to 18 (July 2013).

An editorial honouring his service to the IJSEM was published on the occasion of his retirement [2]. On this occasion, the ICSP honoured him by voting him to the Life Members list.

In 1997, Jean launched the internet site known as LPSN – List of Prokaryotic Names with Standing in Nomenclature [3]. This remarkable database and tool for microbiologists, for which he won several awards, was a work of love on his part and became his legacy; he was its curator until his retirement in 2013. The site was subsequently curated by Aidan C. Parte, and it is now maintained at the Leibniz Institute DSMZ – German Collection of Microorganisms and Cell Cultures in Braunschweig, Germany, and supported by the ICSP, in agreement with the Microbiology Society (UK). The LPSN website (https://lpsn.dsmz.de), which was recognized in December 2023 as a Global Core Biodata Resource, is a primary source for information on taxa of prokaryotes and their nomenclature. Jean added numerous insightful comments to LPSN, which are still available on the website.

Jean also contributed many chapters to *Bergey’s Manual of Systematic Bacteriology* (currently, *Bergey’s Manual of Systematics of Archaea and Bacteria*), and in 2005, he was proud to receive the Bergey’s Award of the Bergey’s Manual Trust for his contribution and service to the community of microbiologists.

The taxa *Euzebya* Kurahashi *et al*. 2010, *Euzebyaceae* Kurahashi *et al*. 2010, *Euzebyales* Kurahashi *et al*. 2010 [4], *Euzebyella* Lucena *et al*. 2010 [5] and *Salinirhabdus euzebyi* Albuquerque *et al*. 2007 [6] were named in honour of Professor Jean Paul Euzéby.

All members of the ICSP celebrate the great career and memory of Professor Euzéby. His outstanding vision, work and dedication to prokaryotic nomenclature have made a lasting impact on the field of Microbiology.

Our heartfelt condolences go out to Professor Euzéby’s family and friends.

## Abbreviations

ENVT: École Nationale Vétérinaire de Toulouse
ICSP: International Committee on Systematics of Prokaryotes
IJSEM: International Journal of Systematic and Evolutionary Microbiology
LPSN: List of Prokaryotic Names with Standing in Nomenclature
